# Adaptive laboratory evolution accelerated glutarate production by *Corynebacterium glutamicum*

**DOI:** 10.1186/s12934-021-01586-3

**Published:** 2021-05-10

**Authors:** Carina Prell, Tobias Busche, Christian Rückert, Lea Nolte, Christoph Brandenbusch, Volker F. Wendisch

**Affiliations:** 1 Genetics of Prokaryotes, Faculty of Biology & CeBiTec, Bielefeld University, Universitätsstr. 25, 33615 Bielefeld, Germany; 2 Technology Platform Genomics, Center for Biotechnology (CeBiTec), Bielefeld University, Sequenz 1, 33615 Bielefeld, Germany; 3 Laboratory of Thermodynamics, Department of Biochemical and Chemical Engineering, TU Dortmund University, Emil-Figge-Str. 70, 44227 Dortmund, Germany

**Keywords:** *Corynebacterium glutamicum*, Glutarate, Adaptive laboratory evolution, Metabolic engineering, Reverse genetics, Volumetric productivity, Reactive extraction

## Abstract

**Background:**

The demand for biobased polymers is increasing steadily worldwide. Microbial hosts for production of their monomeric precursors such as glutarate are developed. To meet the market demand, production hosts have to be improved constantly with respect to product titers and yields, but also shortening bioprocess duration is important.

**Results:**

In this study, adaptive laboratory evolution was used to improve a *C. glutamicum* strain engineered for production of the C_5_-dicarboxylic acid glutarate by flux enforcement. Deletion of the l-glutamic acid dehydrogenase gene *gdh* coupled growth to glutarate production since two transaminases in the glutarate pathway are crucial for nitrogen assimilation. The hypothesis that strains selected for faster glutarate-coupled growth by adaptive laboratory evolution show improved glutarate production was tested. A serial dilution growth experiment allowed isolating faster growing mutants with growth rates increasing from 0.10 h^−1^ by the parental strain to 0.17 h^−1^ by the fastest mutant. Indeed, the fastest growing mutant produced glutarate with a twofold higher volumetric productivity of 0.18 g L^−1^ h^−1^ than the parental strain. Genome sequencing of the evolved strain revealed candidate mutations for improved production. Reverse genetic engineering revealed that an amino acid exchange in the large subunit of l-glutamic acid-2-oxoglutarate aminotransferase was causal for accelerated glutarate production and its beneficial effect was dependent on flux enforcement due to deletion of *gdh*. Performance of the evolved mutant was stable at the 2 L bioreactor-scale operated in batch and fed-batch mode in a mineral salts medium and reached a titer of 22.7 g L^−1^, a yield of 0.23 g g^−1^ and a volumetric productivity of 0.35 g L^−1^ h^−1^. Reactive extraction of glutarate directly from the fermentation broth was optimized leading to yields of 58% and 99% in the reactive extraction and reactive re-extraction step, respectively. The fermentation medium was adapted according to the downstream processing results.

**Conclusion:**

Flux enforcement to couple growth to operation of a product biosynthesis pathway provides a basis to select strains growing and producing faster by adaptive laboratory evolution. After identifying candidate mutations by genome sequencing causal mutations can be identified by reverse genetics. As exemplified here for glutarate production by *C. glutamicum*, this approach allowed deducing rational metabolic engineering strategies.

**Supplementary Information:**

The online version contains supplementary material available at 10.1186/s12934-021-01586-3.

## Introduction

Plastics still are synthesized primarily from natural gas and petroleum and only a fraction of 1% is bio-based. The demand for environmentally friendly alternatives is steadily increasing and the annual market volume of bioplastics is predicted to increase to 18% until 2025 [1]. Biopolyamides are gaining more interest for use in the textile and construction industries. Polyamides can be obtained either by condensation of dicarboxylic acids with diamines or by anionic ring-opening polymerization of lactams, the cyclization products of ω-amino acids [2]. Bio-based production of monomeric building blocks for polyamides has been established in metabolically engineered *C. glutamicum* and *E. coli* [3, 4]. Fermentative production of the C4-ω-amino acid γ-aminobutyrate (GABA) [5, 6] and the C5-ω-amino acid 5-aminovalerate (5AVA) has been established [7, 8] and, e.g., ring-opening polymerization of 5AVA can be used to produce the polyamide 5 (PA 5) [9, 10]. Moreover, diamines like putrescine [11, 12] and cadaverine [13, 14] as well as the dicarboxylic acids succinate and glutarate [15–17] were successfully produced in high titers. Glutarate, e.g., is used as a building block for polyamides such as PA 4.5 [18], PA 6.5, PA 12.5 [19] or PA 5.5 the latter of which  is synthesized by polycondensation of the C5-dicarboxylic acid glutarate with C5-diamine cadaverine [20]. Notably, the C5 polyamide building blocks cadaverine, 5AVA and glutarate can be synthesized from a common precursor, the amino acid l-lysine. Industrial l-lysine production by fermentation with *Corynebacterium glutamicum* is operated at large scale with an annual production volume of about 2.6 million metric tonnes in 2018 [4].

**Fig. 1:**
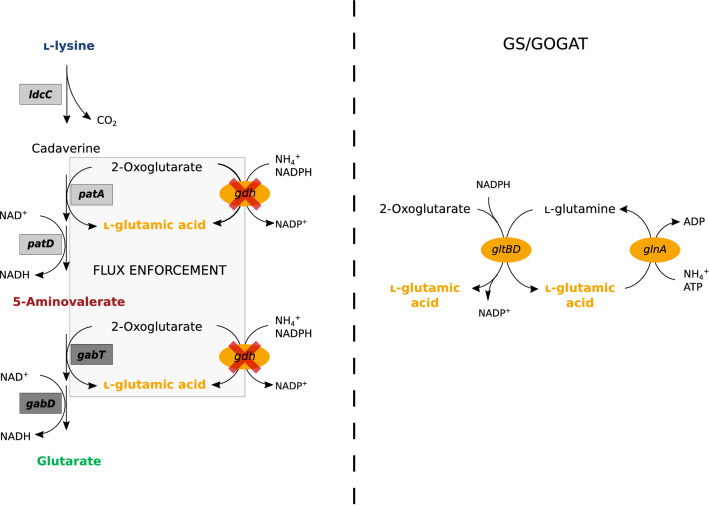
Schematic representation of the metabolic pathway for glutarate production, flux enforcement by deletion of *gdh* (left panel) and ammonium assimilation by the GS/GOGAT system (right panel)*.* Gene names are shown next to enzyme reactions (arrows), gene deletions are indicated by red crosses. Enzymes from *P. stutzeri* (dark grey), *E. coli* (light grey) and native enzymes (orange) are highlighted. *gabT*, GABA/5AVA amino transferase; *gabD*, succinate/glutarate-semialdehyde dehydrogenase; *ldcC*, l-lysine decarboxylase; *patA*, putrescine transaminase; *patD*, γ-aminobutyraldehyde dehydrogenase;*glnA*, glutamine synthetase (GS); *gltBD*, l-glutamic acid-2-oxoglutarate aminotransferase (GOGAT); *gdh*, l-glutamic acid dehydrogenase

Glutarate can be derived from l-lysine by four different pathways. All four pathways converge to 5-aminovalerate (5AVA), which then is converted to glutarate in two enzymatic steps catalyzed by GABA/5AVA aminotransferase (GabT) and succinate/glutarate semialdehyde dehydrogenase (GabD). The first pathway from l-lysine to 5AVA employs l-lysine-α-oxidase (RaiP) from *Scomber japonicus* that catalyzes oxidative deamination of l-lysine using molecular oxygen followed by spontaneous decarboxylation [21]. The second pathway to 5AVA combines oxidative decarboxylation by l-lysine monooxygenase (DavA) using molecular oxygen followed by desamidation by γ-aminovaleramidase (DavB) from *Pseudomonas putida* [20]. The third pathway is based on l-lysine decarboxylase from *E. coli*, putrescine oxidase PuO from *Rhodococcus qingshengii*, which requires molecular oxygen, and γ-aminobutyraldehyde dehydrogenase PatD from *E. coli* [8]. The fourth pathway does not require molecular oxygen as it cascades l-lysine decarboxylase, 2-oxoglurate-dependent putrescine/cadaverine transaminase PatA, and NAD-dependent γ-aminobutyraldehyde dehydrogenase PatD from *E. coli* [7]. The pathway combinations LdcC-PuO-PatD-GabT-GabD and LdcC-PatA-PatD-GabT-GabD couple conversion of l-lysine to glutarate either to one (GabT) or two (PatA, GabT) transaminase reactions, respectively, which generate l-glutamic acid from 2-oxoglutarate. Deletion of *gdh*, the gene for the major ammonium assimilating enzyme l-glutamic acid dehydrogenase [8, 16], enabled flux enforcement (Fig. 1, left panel), i.e., the metabolic setup in which growth requires production of glutarate. In general, GDH is active under nitrogen surplus conditions and has a low affinity towards its substrates ammonia and 2-oxoglutarate [22]. By contrast, the enzyme pair glutamine synthetase (GS) and l-glutamic acid-2-oxoglutarate aminotransferase (GOGAT, also known as l-glutamic acid synthase) synthesizes l-glutamic acid in an ATP dependent manner during ammonium starvation at ammonium concentrations below 5 mM [23]. The GS/GOGAT system is encoded by *glnA* for GS and *gltBD* for the large and small subunits of GOGAT (Fig. 1, right panel). The net reaction of the combined activities of GS and GOGAT results in ATP and NADPH dependent conversion of 2-oxoglutarate to l-glutamic acid, while GDH only requires NADPH for reductive amination of 2-oxoglutarate to l-glutamic acid. It is known that GS/GOGAT can compensate for the lack of GDH [24] also at higher nitrogen concentrations (up to 40 mM) [25].

Systems metabolic engineering proved successful to achieve high titer glutarate production by metabolically engineered *C. glutamicum* [16, 17, 26]. In this study, we aimed to accelerate glutarate production by evolutionary engineering. Adaptive Laboratory Evolution (ALE) allows to leverage natural selection to optimize a target property of a production strain without the requirement of a priori knowledge of the genetic background [27, 28]. This approach is straightforward if a growth advantage can be selected for. This was easily implemented, e.g., when higher tolerance against a compound is sought or to improve substrate utilization and to optimize growth rates [29, 30]. Moreover, it can also be used to identify non‐intuitive targets for strain engineering, and ultimately to gain a comprehensive understanding of biological pathway regulation [31]. In *C. glutamicum*, ALE allowed to accelerate growth of the wild-type [32, 33], to increase tolerance towards higher temperatures [34] and methanol [35, 36], to improve consumption of xylose and cellobiose [37, 38], and to increase production of putrescine and ornithine [39, 40].

We have chosen to apply ALE in order to accelerate glutarate production via the LdcC-PatA-PatD-GabT-GabD pathway since two of the involved transaminase reactions (PatA, GabT) provide l-glutamic acid from 2-oxoglutarate and, thus, compensate for the lack of GDH due to the deletion of its gene [8, 16]. The resulting flux enforcement provides a selectable trait by linking metabolic productivity to growth. In this metabolic setup, the rate of growth (requiring l-glutamic acid) is coupled to the rate of glutarate production (providing l-glutamic acid) and selection of faster growing mutants yielded strains with increased volumetric productivity. Mutations identified by genome sequencing could be rationalized by reverse genetics.

Moreover, in order to complement strain development, purification of glutarate from the fermentation broth using a combination of reactive extraction and reactive re-extraction was considered. For this purpose, we adapted an approach, which was previously used for the purification of itaconic acid and is based on an aqueous organic extraction system and tertiary amines as (reactive) extractants [41], to serve for the recovery of glutarate by identification of optimal (reactive) extractants and organic phases in dedicated screening experiments. During the reactive extraction, the amine extractant interacts with glutarate building a hydrophobic complex, which is then extracted to the organic phase (separation from impurities). The results reveal that the concept allows for an efficient separation of glutarate from a crude fermentation broth showing high yields and selectivities, opening the window for industrial production.

## Results

### Adaptive laboratory evolution of flux enforced glutarate production improved volumetric productivity by metabolically engineered *C. glutamicum*

**Fig. 2:**
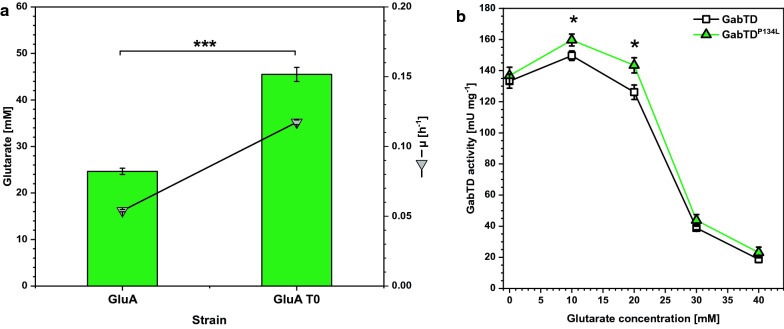
Influence of the amino acid exchange GabD^P134L^ on **a** growth and glutarate production and **b** the combined in vitro enzyme activities of GABA transaminase GabT and succinate semialdehyde dehydrogenase GabD. **a** Strain GluA and GluA T0 were cultivated in the BioLector microcultivation system with 40 g L^−1^ glucose in CGXII minimal medium supplemented with 1 mM IPTG. Supernatant concentrations of glutarate were determined after 96 h and are given as means and standard deviations of three independent cultivations. **b** Crude extracts of DH5α (pEC-XT99A-*gabTD*) and DH5α (pEC-XT99A-*gabTD*^P134L^) were assayed for combined in vitro enzyme activities of GABA transaminase GabT and succinate semialdehyde dehydrogenase GabD with 20 mM 5AVA as substrate and increasing glutarate concentrations. Statistical significance was assessed in Student’s unpaired *t*-test (*p < 0.05)

By serendipity, we found that repeated cultivation of strain GluA [16] starting from single colonies yielded a variant that showed faster growth (named GluA T0). Plasmid insert sequencing revealed that a point mutation occurred in the gene coding for succinate semialdehyde dehydrogenase GabD from *P. stutzeri*. This resulted in amino acid exchange P134L in GabD. The maximal growth rate of glutarate producer GluA T0 with GabD^P134L^ was increased to 0.12 ± 0.00 h^−1^ in comparison to 0.05 ± 0.00 h^−1^ for the isogenic strain with wild-type GabD (Fig. 2a). Since growth was coupled to production by flux enforcement, an almost twofold higher glutarate production resulted using GabD^P134L^ (45 ± 2 mM) instead of the native version of GabD (25 ± 1 mM). A Phyre model of the succinate semialdehyde dehydrogenase GabD from *P. stutzeri* [22] revealed that P134 is part of the oligomerization interface of the protein [42]. GabD from *P. putida* [43] and *E. coli* [44] are homotetramers. CUPSAT calculations of GabD^P134L^ suggested decreased protein stability as compared to GabD [45]. This prompted us to determine GabD activities in crude extracts of *E. coli* strains DH5α (pEC-XT99A-*gabTD*) and DH5α (pEC-XT99A-*gabTD*^P134L^). The combined in vitro enzyme activities of GABA transaminase GabT and GabD were monitored spectrophotometrically. To study if the amino acid exchange P134L affects a possible feedback inhibition by glutarate, these assays were performed in the presence of glutarate concentrations up to 40 mM. Feedback-inhibition by 30 and 40 mM glutarate was found for both GabD and GabD^P134L^ (Fig. 2b), while at 10 mM glutarate their activities were increased. Notably, in the presence of 10 and 20 mM glutarate GabD^P134L^ activity was significantly higher than that of GabD (Fig. 2b).

**Fig. 3:**
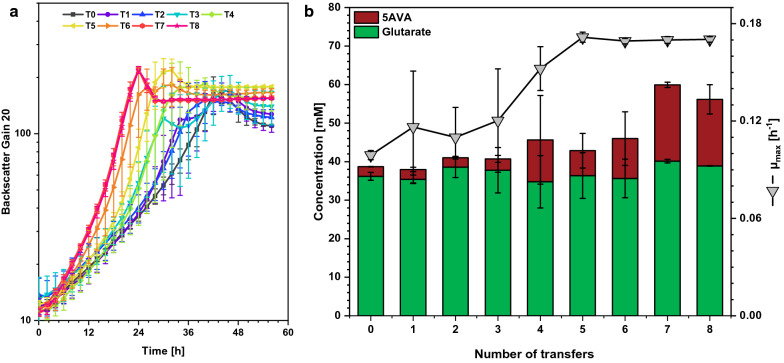
Characterization of the evolved strains after eight transfers (T0–T8) regarding **a** growth and **b** production of glutarate and 5AVA. Cells were grown in the BioLector microcultivation system using 40 g L^−1^ glucose minimal medium supplemented with 1 mM IPTG and harvested after 56 h. Values and error bars represent mean and standard deviation values (n = 3 cultivations)

This initial finding of a mutation of the original strain that led to faster growth and better glutarate production prompted us to perform an ALE experiment. Therefore, strain GluA T0 was used to start a serial transfer and after it grew from the OD_600_ of 1 at inoculation to an OD_600_ of approximately 8. At every transfer, one aliquot was streaked out on agar plates and three single colonies were selected and used to make glycerol stocks (triplicate glycerol stocks GluA T1 until triplicate glycerol stocks GluA T8). A second aliquot was used to inoculate the subsequent transfer culture to an initial OD_600_ of 1. This process was repeated until the eighth transfer culture (GluA T8) and times to reach an OD_600_ about 8 were shortened from transfer to transfer. For comparison, a growth and glutarate production experiment was performed in the BioLector microcultivation system starting from the frozen glycerol stocks of strains GluA T0 to GluA T8. Confirming the observations during the ALE transfer experiment, growth of the evolved strains gradually accelerated (Fig. 3a). The characterization of the evolved strains revealed that the maximal growth rate µ_max_ increased gradually from 0.10 h^−1^ for the parental glutarate producer (GluA T0) to 0.17 h^−1^ for GluA T5, but it did not increase further for the following three transfers (Fig. 3b).

As growth was coupled to production by flux enforcement, glutarate productivity increased when growth was accelerated (Fig. 3b). The maximal product titers of glutarate and its precursor 5AVA were reached after the seventh transfer and ALE strain GluA T7 accumulated 10% more glutarate and tenfold more 5AVA (20 ± 1 mM compared to 2 ± 0 mM) than the parent strain (Fig. 3b). As consequence of the combined beneficial effects of faster growth and increased production, ALE strain GluA T7 reached its maximal biomass already after 24 h (Fig. 3a). The volumetric productivity of 0.18 ± 0.00 g L^−1^ h^−1^ was twofold higher than that of the parental GluA T0 (0.09 ± 0.00 g L^−1^ h^−1^). GluA T5 had its maximum OD_600_ after 30 h with a volumetric productivity of 0.13 ± 0.00 g L^−1^ h^−1^.

### RNAseq analysis of global gene expression patterns in ALE strains GluA T5 and GluA T7

The ALE experiment allowed to select strain GluA T5, which grew faster and produced more glutarate than the parent strain, and strain GluA T7 that grew as fast as GluA T5, but which produced more 5AVA than GluA T5. In order to reveal gene expression changes between GluA T5, GluA T7 and the parental strain GluA T0, RNA was isolated from cells growing exponentially in CGXII minimal medium supplemented with 40 g L^−1^ glucose and 1 mM IPTG in triplicates, sequenced and mapped to the reference genome (Additional file 2: Tables S1, S2). Of the 110 genes differentially expressed in the comparison between strains GluA T5 and GluA T0, 52 genes were downregulated in GluA T5. Among them, *sucCD* (cg2835, cg2837), coding for succinyl-CoA synthetase, and *aceA* (cg2560), encoding isocitrate lyase, are key enzymes of the TCA cycle and the glyoxylate shunt. Of the 62 genes upregulated in GluA T5, seven code for transporters, e.g., mediating uptake of phosphate (cg1651), Na^+^ (cg1624, cg3027, cg3028), Co^2+^ (cg3134) or iron (cg0770, cg0771), and one for an uncharacterized transcriptional regulator (cg3291). Since flux enforcement involved deletion of the dehydrogenase gene *gdh*, it is noteworthy that the gene for the putative l-glutamic acid exporter YggB (cg1434) was downregulated in both GluA T5 and GluA T7.

Of the 173 genes differentially expressed in the comparison between GluA T7 and the parental strain GluA T0, 107 were downregulated in GluA T7. Many of those genes are involved in the nitrogen starvation response and they belong to AmtR regulon: *ureABCEFGD* (encoding urease), *urtABCDE* (encoding ABC-transporter for urea), *amt-ocd-soxA* (coding for ammonium permease, ornithine decarboxylase and sarcosine oxidase), *amtB-glnK-glnD* (ammonium transporter, GlnK, uridylyltrasnferase) and *gltBD* (glutamine 2-oxoglutarate amidotransferase) [22, 46]. Thus, the gene expression changes observed in ALE strains GluA T5 and GluA T7 affected nitrogen assimilation, l-glutamic acid metabolism and transport processes, but not genes of the synthetic LdcC-PatA-PatD-GabT-GabD pathway for glutarate production. At first sight, the transcriptome analysis was not instructive to explain the improved glutarate production of the strains selected by ALE.

### Genome sequencing and reverse genetics for identification of mutations causal for improved glutarate production by ALE strains GluA T5 and GluA T7

The genomes of the ALE strains GluA T5, which grew faster and produced more glutarate than the parental strain, and GluA T7 that did not grow faster than GluA T5, but produced more 5AVA and glutarate than GluA T5, were sequenced to identify genetic differences to the parental strain. Subsequently, reverse engineering was performed to identify those mutations that are causal for improved growth and/or glutarate production.

Surprisingly, genome sequencing of strain GluA T5 did neither reveal genome-based nor plasmid-based mutations in comparison to the non-evolved strain GluA T0. However, analysis of the sequence reads mapped to DNA of the three expression plasmids showed increased plasmid copy numbers (PCN) of all three plasmids. In the absence of changes in the DNA sequence of the plasmids, we can only invoke epigenetic changes, e.g. due to methylation. It is known that plasmid DNA methylation is required for plasmid replication contributing to regulation of replication reinitiation and the accuracy of the copy-number control [47]. The PCN of plasmid pVWEx1-*ldcC* carrying the gene for the first step of glutarate production was 13 ± 1, and 17 ± 1 for strains GluA T0 and GluA T5, respectively. For plasmid pEKEx3-*patDA* comprising genes coding for step two and three of the pathway the PCN increased from 107 ± 2 (GluA T0) to 121 ± 11 (GluA T5). The PCN for the plasmid pEC-XT99A-*gabTD*^P134L^ with the genes of the last two steps was 10 ± 1 for GluA T0 and 14 ± 0 for GluA T5. Taken together, genome sequencing revealed only increased PCN for all three plasmids that provide the glutarate biosynthesis pathway and this may explain faster growth of GluA T5 as compared to GluA T0.

Genome sequencing of strain GluA T7 revealed comparable PCN for plasmids pVWEx1-*ldcC* (17 ± 1 vs. 17 ± 1) and pEKEx3-*patDA* (124 ± 2 vs. 121 ± 11) as compared to GluA T5, while the PCN of plasmid pEC-XT99A-*gabTD*^P134L^ was higher (19 ± 0 vs. 14 ± 0). Importantly, two mutations were identified in GluA T7 that were absent from strains GluA T0 and GluA T5. The first mutation was found in plasmid pEC-XT99A-*gabTD*^P134L^: a deletion of 21 bp in the coding sequence of the antibiotic resistance marker of this plasmid, i.e. in for tetracycline efflux permease gene *tetA(Z)*, shortened the encoded protein by 7 amino acids. The deletion comprised a direct repeat (5′-TGACTGCTCGCTACTCTCATC-3′ (*tetA(Z)*^Δ21bp^, Additional file 1: Figure S1A). As consequence the TetZ protein was shortened and lacked amino acids 8 to 14 in the N-terminal part before the first transmembrane helix. RNAseq data confirmed the deletion and showed that the mutated gene was transcribed. Analysis of the shortened protein by SignalP [48] and by TMHMM [49] suggested that there is no signal peptide and predicted twelve transmembrane helices as in the intact tetracycline efflux permease protein, thus, the sequence is shortened before the first transmembrane helices of the protein. Deletion of direct repeats on plasmids are known and the frequency of these events depends on the location and the length of the repeats [50, 51]. Moreover, the absence of the antibiotic resistance and the growth rate are crucial for the frequency [52]. To investigate the influence of shortage on the protein function, we determined the minimum inhibitory concentration (MIC) for tetracycline. GluA T0 displayed a MIC of 20 µg mL^−1^ tetracycline, whereas a strain carrying the mutated plasmid only had a MIC of 10 µg mL^−1^ (Additional file 1: Figure S1B). Possibly, the higher PCN of plasmid pEC-XT99A-*gabTD*^P134L^ in GluA T7 as compared to GluA T0 and GluA T5 compensated for the reduced antibiotic resistance level by mutation pEC-XT99A-*tetA(Z)*^Δ21bp^.

The second mutation affected an enzyme involved in l-glutamic acid biosynthesis from 2-oxoglutarate, glutamine 2-oxoglutarate aminotransferase (Fig. 1). This single nucleotide polymorphism (SNP) was named *gltB*^E686Q^ as it resulted in an exchange of amino acid 686 in the large subunit of glutamine 2-oxoglutarate aminotransferase GltB from l-glutamic acid (E) to glutamine (Q). Analysis of the 3D structure of GltB by Phyre2 [42] did neither suggest that the active center nor the binding of the small subunit GltD was affected. CUPSAT analysis [45] suggested that this amino acid exchange may have a destabilizing effect. COACH-D based modeling [53] did not indicate altered interaction with the substrates 2-oxoglutarate and NADPH, but may indicate changed binding of the substrate glutamine. The determination of the *K*_m_ for glutamine in the crude extract of GRLys1Δ*gltB* overexpressing the native and the mutated version of *gltBD* revealed that the SNP in GltB resulted in a twofold higher *K*_m_ of 0.52 ± 0.04 mM than the wild-type enzyme (0.25 ± 0.03 mM) (Additional file 1: Figure S2). The wild-type *K*_m_ was comparable to that reported for the related *C. glutamicum* subsp. *flavum* (0.24 mM) [54].

To test if the identified mutations are causal for improved glutarate production, they were introduced into the parental strain GluA T0 either individually or combined. First, the mutated vector named pEC-XT99A-*tetA(Z)*^Δ21bp^-*gabTD*^P134L^ was isolated from the evolved strain GluA T7 and used to replace the unmutated vector pEC-XT99A-*gabTD*^P134L^ in GluA T0. To this end, pEC-XT99A-*tetA(Z)*^Δ21bp^-*gabTD*^P134L^ was used to transform the precursor strain GSLA2G (pVWEx1-*ldcC*) (pEKEx3-*patDA*) and the obtained strain was named GluA RG1. Next, the SNP *gltB*^E686Q^ was introduced into GluA T0 (via two-step recombination in the genome of the plasmid-less precursor strain GSLA2G, followed by transformation with the plasmids pVWEx1-*ldcC*, pEKEx3-*patDA* and pEC-XT99A-*gabTD*^P134L^) yielding strain GluA RG2. Strain GluA RG3 was constructed to contain both, the SNP *gltB*^E686Q^ and the plasmid pEC-XT99A-*tetA(Z)*^Δ21bp^-*gabTD*^P134L^.

Strain GluA RG1 grew very slowly (0.04 ± 0.01 h^−1)^ to a reduced biomass concentration and glutarate production (23 ± 1 mM) was reduced to half as compared to the parental strain GluA T0 (45 ± 2 mM). However, the glutarate yield per biomass of 1.2 g g^−1^ was comparable to that of GluA T0 (1.0 g g^−1^) (Table 1). Thus, as a result of the 21 bp off-frame deletion in the coding sequence of *tetA(Z)* in plasmid pEC-XT99A-*tetA(Z)*^Δ21bp^-*gabTD*^P134L^, growth was perturbed, while the cell-specific glutarate production was not affected.

**Table 1:** Effect of the SNP *gltB*^E686Q^ and the plasmid pEC-XT99A-*tetA(Z)*^Δ21bp^-*gabTD*^P134L^ on biomass formation (CDW), substrate specific yield (Y_P/S_), biomass specific yield (Y_P/X_) and volumetric productivity (VP)

Strain	CDW (g L^−1^)	Y_P/S_ (g g^−1^)	Y_P/X_ (g g^−1^)	VP (g L^−1^ h^−1^)
GluA T0	6.2 ± 0.6	0.15 ± 0.01	1.0 ± 0.1	0.13 ± 0.00
GluA T7	6.5 ± 0.3	0.16 ± 0.00	1.0 ± 0.0	0.24 ± 0.00
GluA RG1	2.6 ± 0.1	0.08 ± 0.00	1.1 ± 0.0	0.03 ± 0.00
GluA RG2	6.5 ± 0.5	0.18 ± 0.01	1.1 ± 0.0	0.14 ± 0.00
GluA RG3	6.4 ± 0.3	0.18 ± 0.00	1.2 ± 0.1	0.19 ± 0.00

**Fig. 4:**
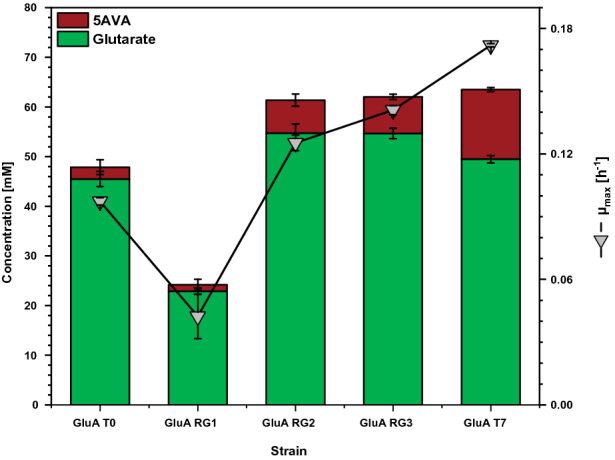
Influence of the SNP *gltB*^E686Q^ and the plasmid pEC-XT99A-*tetA(Z)*^Δ21bp^-*gabTD*^P134L^ on maximal growth rate, and glutarate and 5AVA production by stepwise reverse genetics. Cells were grown in the BioLector microcultivation system using 40 g L^−1^ glucose minimal medium supplemented with 1 mM IPTG and harvested after 96 h. Values and error bars represent mean and standard deviation values (n = 3 cultivations)

As consequence of introducing the amino acid exchange E686Q in GltB, strain GluA RG2 produced more glutarate (55 ± 2 mM; + 22%) and more of its direct precursor 5AVA (7 ± 1 mM; + 250%; Fig. 4) as compared to the parental GluA T0. Due to flux enforcement, strain GluA RG2 also grew faster than GluA T0 (0.13 h^−1^; + 30%). This result is commensurate with the finding by RNAseq analysis that the nitrogen starvation response is triggered in strains GluA T0 and GluA T5 that lack the amino acid exchange E686Q in GltB, but not in GluA T7 that carries this mutation.

The reverse engineered strain GluA RG3 that carried the 21 bp deletion in pEC-XT99A in addition to GltB^E686Q^ showed a higher growth rate (Fig. 4). Since the glutarate titer was comparable for GluA RG2 and GluA RG3, a higher volumetric productivity for glutarate resulted when mutations GltB^E686Q^ and the 21 bp deletion were both present (Table 1). Thus, GltB^E686Q^ and the 21 bp deletion showed covariance with the beneficial effect on glutarate by GltB^E686Q^ being epistatic over the 21 bp deletion. In other words, this may indicate that only after GltB^E686Q^ improved l-glutamic acid biosynthesis, the 21 bp deletion supported faster growth and, thus, higher volumetric productivity for glutarate.

### The beneficial effect of GltB^E686Q^ depends on the deletion of *gdh*

**Fig. 5:**
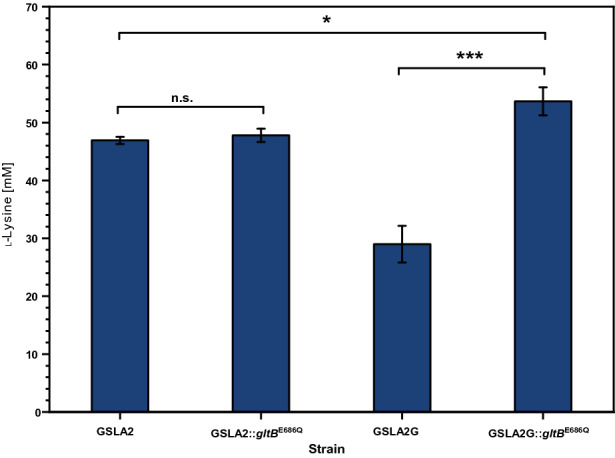
Influence of the SNP GltB^E686Q^ on l-lysine production in dependency of the *gdh* deletion. Cells were grown in the BioLector microcultivation system using 40 g L^−1^ glucose minimal medium and harvested after 48 h. Values and error bars represent mean and standard deviation values (n = 3 cultivations). Statistical significance was assessed in Student’s unpaired *t*-test (***p < 0.001, *p < 0.05, n.s. not significant)

Next, we tested if the beneficial effect of GltB^E686Q^ requires (a) deletion of *gdh* or (b) is only seen upon flux enforced glutarate production via the LdcC-PatA-PatD-GabT-GabD route. Therefore, the GltB^E686Q^ mutation was introduced either into the plasmid-less precursor strain GSLA2, which overproduces l-lysine, or its derivative GSLA2G that carries the *gdh* deletion. Production of l-lysine by these strains, named GSLA2::*gltB*^E686Q^ and GSLA2G::*gltB*^E686Q^, was compared to l-lysine production by their parental strains GSLA2 and GSLA2G that carry wild-type *gltB*. As consequence of the *gdh* deletion*,* production of l-lysine was decreased by about 38% (compare strains GSLA2 and GSLA2G in Fig. 5). While deletion of *gdh* is known to be dispensable for biosynthesis of L-glutamic acid for growth requirements [24], deletion of *gdh* was negative for overproduction of lysine (Fig. 5). This indicated that the GS/GOGAT system suffices to replace *gdh* for growth requirements, but not to provide enough assimilated nitrogen for overproduction of the amino acid l-lysine. Introduction of the GltB^E686Q^ mutation into strain GSLA2 did not change l-lysine production significantly (compare strain GSLA2::*gltB*^E686Q^ with GSLA2 in Fig. 5). Upon introduction of the GltB^E686Q^ mutation into the *gdh* deletion mutant GSLA2G, significantly more l-lysine was produced (compare strain GSLA2G::*gltB*^E686Q^ with GSLA2G in Fig. 5). This indicated that provision of assimilated nitrogen via the mutant GS/GOGAT system in the *gdh* deletion mutant was apparently high enough to support production of l-lysine to a titer comparable to that of the *gdh*-positive parental strain GSLA2. Thus, the beneficial effect of GltB^E686Q^ is dependent on deletion of *gdh*.

Moreover, since the combined presence of the GltB^E686Q^ mutation and the *gdh* deletion improved both production of l-lysine (Fig. 5) and of glutarate (Fig. 4), the beneficial effect of GltB^E686Q^ is independent of conversion of l-lysine to glutarate via the LdcC-PatA-PatD-GabT-GabD route. This mutation pair (*gltB*^E686Q^, Δ*gdh*) may be helpful for production processes that involve l-lysine as intermediate.

### Fermentative glutarate production by evolved strain GluA T7 in comparison to GluA T0

**Fig. 6:**
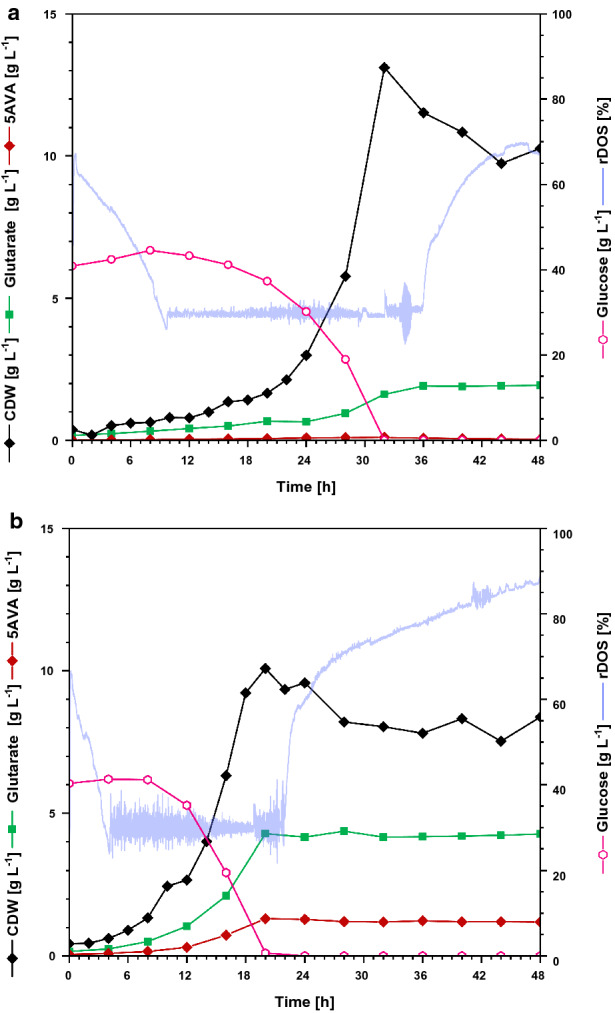
Glutarate production by *C. glutamicum* GluA T0 (**a**) and GluA T7 (**b**) in bioreactors operated in batch mode. Both strains were cultivated in CGXII minimal medium in batch mode over 48 h, containing 40 g L^−1^ glucose. Glutarate production is indicated in green squares (g L^−1^), biomass concentration (CDW) is shown in black diamonds (g L^−1^), glucose concentration (g L^−1^) is plotted as pink hollow triangles, and 5AVA concentration (g L^−1^) in red diamonds and the relative dissolved oxygen saturation (rDOS) is indicated in light blue (%). Cultivation was performed at 30 °C and pH 7.0 regulated with 10% (*v/v*) H_3_PO_4_ and 4 M KOH. 0.6 mL L^−1^ of antifoam agent AF204 (Sigma Aldrich, Taufkirchen, Germany) was added to the medium manually before inoculation

Since stable performance of a production host in reactor scale is essential for the biotechnological application, we compared the evolved strain GluA T7 with the parental strain GluA T0 in batch-mode bioreactor cultivation (3.7 L). The parental strain GluA T0 grew with a maximal growth rate of 0.11 h^−1^ and reached its maximal biomass concentration of 13.1 g L^−1^ after 32 h (Fig. 6a). The final titer was 2.0 g L^−1^ glutarate and remained stable after 36 h of cultivation. The volumetric productivity was 0.05 g L^−1^ h^−1^, the biomass-specific yield of 0.19 g (g CDW)^−1^ and the substrate-specific yield 0.04 g g^−1^. By comparison, GluA T7 grew with a growth rate of 0.19 h^−1^ and reached its maximal biomass of 10.1 g L^−1^ already after 20 h cultivation (Fig. 6b). The glutarate titer was 4.3 g L^−1^ and 1.2 g L^−1^ 5AVA accumulated as by-product. The volumetric productivity was 0.21 g L^−1^ h^−1^ with a substrate-specific yield of 0.10 g g^−1^ and a biomass-specific yield of 0.51 g g^−1^. Thus, in batch-mode fermentation, GluA T7 clearly outcompeted its precursor strain regarding product titer, volumetric productivity, and yield (Fig. 6).

**Fig. 7:**
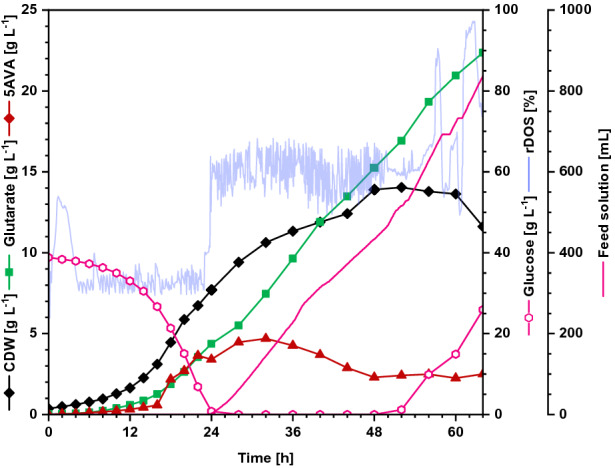
Glutarate production by *C. glutamicum* GluA T7 in fed-batch mode. GluA T7 was cultivated in CGXII minimal medium in fed-batch mode over 64 h, containing 40 g L^−1^ glucose and 150 g L^−1^ glucose from the feeding solution. Glutarate production is indicated in green squares (g L^−1^), biomass concentration (CDW) is shown in black diamonds (g L^−1^), glucose concentration (g L^−1^) is plotted as pink hollow triangles, and 5AVA concentration (g L^−1^) in red diamonds, feed solution (mL) is plotted as pink line and the relative dissolved oxygen saturation (rDOS) is indicated in light blue (%). Cultivation was performed at 30 °C and pH 7.0 regulated with 10% (*v/v*) H_3_PO_4_ and 4 M KOH. An overpressure of 0.4 bar was applied. 0.6 mL L^−1^ of antifoam agent AF204 (Sigma Aldrich, Taufkirchen, Germany) was added to the medium manually before inoculation

Next, fed-batch mode cultivation of GluA T7 was performed to achieve higher glutarate titers. In the batch phase (0–24 h), the cells grew with a growth rate of 0.15 h^−1^ up to 7.7 g L^−1^ cell dry weight (CDW) and 4.4 g L^−1^ glutarate was produced (Fig. 7). After 24 h the feed was started and in the following 40 h around 890 mL feed solution (ρ = 1.1 kg m^−3^) were added. In total, 22.7 g L^−1^ of glutarate were produced within 64 h with a volumetric productivity of 0.35 g L^−1^ h^−1^. The glutarate yield on glucose was 0.23 g g^−1^. With a maximal biomass concentration of 14.0 g L^−1^, 1 g cells produced 1.64 g glutarate.

### Reactive extraction of glutarate from the cultivation broth

After strain engineering enabled an efficient bioreactor process, we sought to develop efficient downstream processing for glutarate. To this end, reactive extraction experiments were conducted with molar ratios of amine extractant/glutarate ranging from 1/1 to 10/1 in order to investigate the required amount of amine extractant for an optimal reactive extraction (maximizing the yield). Amine extractants screened for this purpose were tri-n-hexylamine (T-C6) and tri-n-octylamine (T-C8) and ethyl oleate was chosen as organic phase due to its hydrophobic nature and biocompatibility [55].

**Fig. 8:**
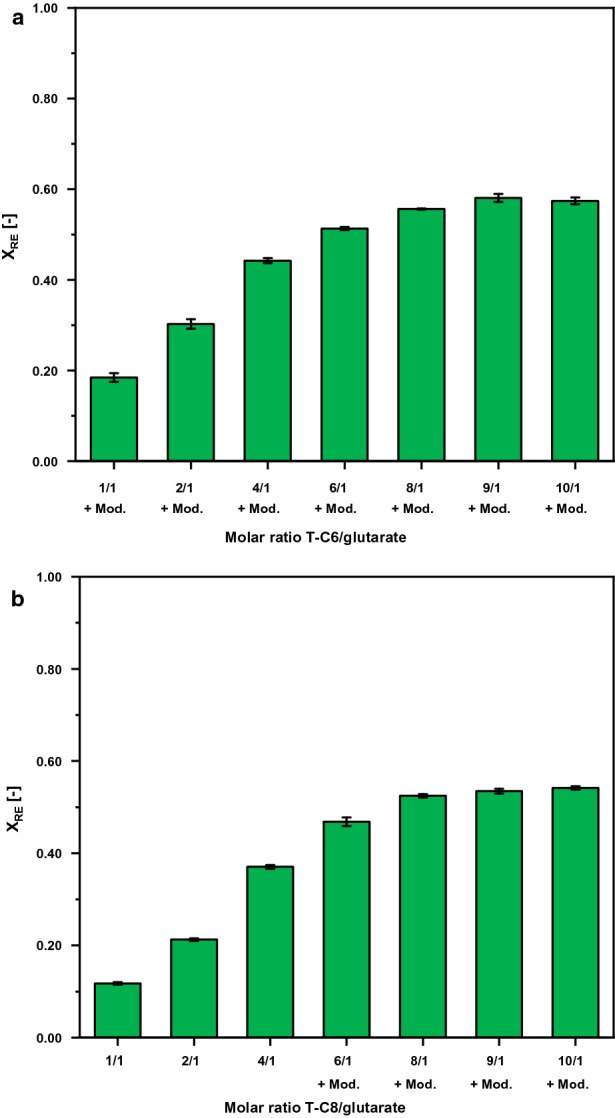
Reactive extraction yields of glutarate from fermentation broth at *T* = 25 °C and p = 1 bar using either T-C6 (**a**) or T-C8 (**b**) as amine extractant. The pH of the fermentation broth was adjusted to 2.5 using highly concentrated sulfuric acid before reactive extraction. **a** T-C6 was used as amine extractant in the organic solvent ethyl oleate at molar ratios of T-C6/glutarate ranging from 1/1 to 10/1. 10 wt% of 1-dodecanol were added to the organic phase as modifier. **b** T-C8 was used as amine extractant in the organic solvent ethyl oleate at molar ratios of T-C8/glutarate ranging from 1/1 to 10/1. Systems with a molar ratio of T-C8/glutarate = 6/1 or higher contained 10 wt% 1-dodecanol as modifier in the organic solvent (+Mod.)

If T-C6 was used as (reactive) amine extractant in combination with ethyl oleate organic phase, the formation of a third-phase was observed for all amine concentrations applied. To circumvent the formation of a third phase, 1-dodecanol was added to the organic extraction phase as (polar) modifier to increase the polarity of the organic phase, and, thus, to enhance the solubility of the hydrophobic acid–amine complex in the organic phase. The optimal amount of modifier in the organic phase, i.e., the lowest amount necessary to prevent third-phase formation, was identified to be 10 wt% for the reactive extraction system containing T-C6 (ratio of amine extractant/glutarate = 10/1). The results of reactive extraction experiments at *T* = 25 °C and *p* = 1 bar using T-C6 as amine extractant in ethyl oleate containing 10 wt% of 1-dodecanol are illustrated in Fig. 8a.

The results clearly showed that with increasing T-C6 content, the amount of glutarate being extracted from the aqueous phases increased until reaching a plateau at 58.1% glutarate yield (for molar ratios of T-C6/glutarate = 9/1 or higher). In contrast to T-C6 as amine extractant, reactive extraction systems containing T-C8 as amine extractant only showed a formation of a third-phase at molar ratios of T-C8/glutarate = 6/1 or higher. Thus, 1-dodecanol was used as polar modifier (10 wt% 1-dodecanol in the organic phase) for extractions at molar ratios exceeding 6/1. The results of reactive extraction experiments at *T* = 25 °C and *p* = 1 bar using T-C8 as amine extractant are shown in Fig. 8b. The yield increased with increasing amount of T-C8 in the organic phase reaching a near plateau for high ratios of T-C8/glutarate, namely 8/1 and 10/1, where yield only increased from 52.5 to 54.1%. As higher reactive extraction yields could be achieved using T-C6 at a lower molar ratio of 9/1 (up to 58.1%), higher molar ratios of T-C8 (exceeding 10/1) were not investigated. As a result of these experiments, the system containing T-C6 as amine extractant, ethyl oleate as organic phase containing 10 wt% of 1-dodecanol as polar modifier, served as basis for the following re-extraction experiments described hereafter.

**Fig. 9:**
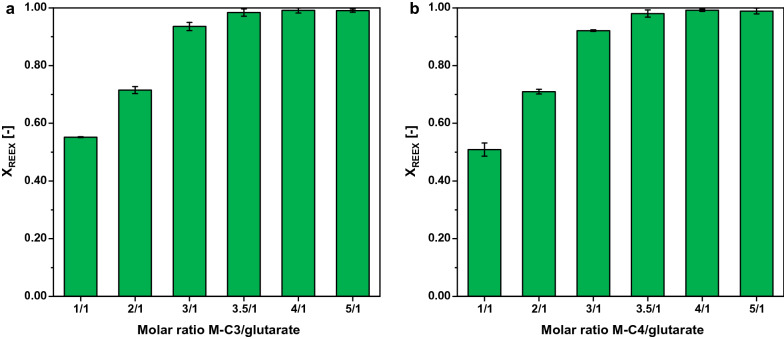
Re-extraction yields of glutarate from the organic phase after reactive extraction using T-C6 (T-C6/glutarate = 9/1) in ethyl oleate (containing 10 wt% of 1-dodecanol) at *T* = 25 °C and *p* = 1 bar. The water-soluble amines **a** M-C3 and **b** M-C4 were used for re-extraction at molar ratios of WSA/glutarate (organic) ranging from 1/1 to 5/1

Upon reactive re-extraction, the acid–amine complex in the organic phase is brought into contact with a water-soluble amine (WSA) in a (fresh) aqueous phase. As the WSA forms a stronger complex with the acid, the acid is displaced from the organic to the aqueous phase forming a water-soluble complex with the WSA. The results for re-extraction experiments conducted with the water-soluble amines n-propylamine (M-C3) and n-butylamine (M-C4) at molar ratios of WSA/glutarate (organic) ranging from 1/1 to 5/1 at *T* = 25 °C and *p* = 1 bar are illustrated in Fig. 9. Increasing amounts of both WSA’s led to increasing re-extraction yields until the maximum respective re-extraction yield was reached in systems containing a molar ratio of WSA/glutarate (organic) = 3.5/1 or higher. The use of both WSA’s as amine extractant led to maximum re-extraction yields of 99%. Taken together, efficient downstream processing of glutarate containing fermentation broth based on a combination of reactive extraction and reactive re-extraction of glutarate was developed.

## Discussion

In this study, flux enforcement coupling growth to l-glutamic acid production by *C. glutamicum* provided the basis to select strains growing and producing faster by adaptive laboratory evolution. Among the candidate mutations determined by genome sequencing two causal mutations were identified by reverse genetics. This approach almost doubled volumetric productivity and in fed-batch bioreactor cultures a titer of 22.7 g L^−1^, a yield of 0.23 g g^−1^ and a volumetric productivity of 0.35 g L^−1^ h^−1^ were achieved. Purification of glutarate directly from the fermentation broth leading to yields of 58% and 99% in the reactive extraction and reactive re-extraction step, respectively, was established.

This report is not the first on metabolic engineering of *C. glutamicum* for glutarate production [8, 16, 17, 26], but the first example of improving volumetric productivity by flux enforcement and ALE. Notably, engineering of the GS/GOGAT system, which was identified here as crucial to accelerate glutarate production, has not been reported previously as metabolic engineering target for glutarate production by *C. glutamicum*. Strain engineering, e.g., by overexpressing *ynfM* encoding the recently discovered glutarate exporter, media optimization, e.g., by using mixtures of glucose and sucrose, and process intensification, e.g., by using a pH–stat feeding strategy in fed-batch cultures, have been described to boost glutarate production to titers of more than 100 g L^−1^ [17]. Thus, the mutation pair identified here (GltB^E686Q^ and deletion of *gdh*) complements the previously described metabolic engineering strategies for glutarate production by *C. glutamicum*. It has to be noted that *yggB* RNA levels were reduced in both ALE strains and this gene codes for a transport system involved in export of l-glutamic acid out of the *C. glutamicum* cell [56], although l-glutamic acid export is not abolished in its absence [57]. Since the flux enforcement strategy used here relied on *gdh* deletion, thus, the major enzyme for synthesis of l-glutamic acid is absent, the reduced *yggB* RNA levels may help to avoid loss of l-glutamic acid from the *C. glutamicum* cell by export. Possibly, deletion of *yggB* may increase stringency of the flux enforcement by *gdh* deletion. Transport engineering has proven important for improving *C. glutamicum* processes [58], not only regarding substrate uptake [59] or product export (e.g., *ynfM*, [17]), but also to avoid loss of intermediates (used here to avoid export of the intermediate cadaverine).

The strains analysed here lacked *gdh*. The growth rate of a *gdh* deletion mutant is reduced due to a partially triggered nitrogen starvation response as evidenced by, e.g., partial adenylylation of GlnK, such that ammonium is assimilated by GS/GOGAT. However, since the intracellular l-glutamic acid pool is not completely restored and the GS/GOGAT has an increased energy demand (1 ATP per NH_3_ fixed), growth in the absence of *gdh* is slower than in its presence. The SNP in *gltB* resulted in amino acid exchange E686Q in the large subunit of GOGAT (GltB^E686Q^). The large subunit serves two functions allocated to two domains: hydrolysis of l-glutamine to NH_3_ and l-glutamic acid on the one hand and combining the produced NH_3_ with 2-oxoglutarate to produce a second molecule of l-glutamic acid on the other hand. The small subunit transfers electrons from the co-substrate NADPH. The SNP may interfere with binding of glutamine according to our inspection of the structure by Phyre2, CUPSAT and COACH-D [42, 53, 60]. Currently, it is unknown how the amino acid exchange E686Q in the large subunit of GOGAT supports faster glutarate production by the strains analysed here. Since it is known that at high NH_3_ concentrations (up to 40 mM) GS/GOGAT compensates for the lack of GDH [25], it is conceivable that the GOGAT mutant GltB^E686Q^ is active at higher nitrogen concentrations. The media used here contains 468 mM NH_3_, which is particularly relevant in the early growth and production phase. RNAseq analysis revealed higher expression of genes belonging to the AmtR regulon of nitrogen starvation in GluA T5 and the parental strain GluA T0 than in GluA T7, which carries GltB^E686Q^ (Additional file 2: Tables S1 and S2). Binding to operator DNA by homodimeric AmtR is not released by a small-molecule effector, but by GlnK when adenylylated at tyrosine residue 51 [61], likely in a 6:6 stoichiometric (AmtR_2_)_3_–(GlnK_3_)_2_ complex [62]. The adenylation status of PII-type signal transduction protein GlnK is controlled by adenyltransferase GlnD under nitrogen starvation, which is most likely perceived as ammonium limitation [63]. Thus, ammonium starvation is less pronounced or less perceived in the GltB^E686Q^ carrying mutant GluA T7 than in the native GltB carrying strains GluA T0 and GluA T5. Besides possible effects due to transcriptional and post-translational regulation, the higher *K*_m_ of GltB^E686Q^ for glutamine as compared to native GltB may provide a clue why GltB^E686Q^ performed better regarding glutarate production. GOGAT competes with the transaminases GabT and PatA for the substrate 2-oxoglutarate, and according to the BRENDA database the *K*_m_ for 2-oxoglutarate is lower for GOGAT from *C. glutamicum* subsp. *flavum* (0.06 mM) than for GabT from *P. aeruginosa* (0.75 mM) and PatA from *E. coli* (19 mM). Thus, these kinetic parameters suggest that GOGAT may outcompete the transaminases used in the synthetic glutarate pathway. The variant GltB^E686Q^ selected by ALE showed a two-fold higher *K*_m_ for glutamine, the other substrate of GOGAT. Thus, this mutation likely favors a higher ratio of 2-oxoglutarate conversion via the transaminases, which is in line with the observed increase in glutarate productivity. However, it has to be noted that while nitrogen-replete *E. coli* cells show relatively low intracellular glutamine (0.2 to 0.5 mM) and 2-oxoglutarate (0.1 to 0.9 mM) concentrations [64], *C. glutamicum* has been described to accumulate about 10 mM glutamine under nitrogen-replete conditions [65]. Future research will have to unravel the mechanism and to distinguish whether nitrogen metabolism and/or regulation is altered due to GltB^E686Q^.

Two changes affected expression and activities of the heterologous 5AVA amino transferase GabT and glutarate-semialdehyde dehydrogenase GabD: the amino acid exchange P134L in GabD led to higher combined GabD-GabT in vitro activity in crude extracts and a higher PCN of plasmid pEC-XT99A-*tetA(Z)*^Δ21bp^-*gabTD*^P134L^ that differed from pEC-XT99A-*gabTD*^P134L^ by a 21 bp off-frame deletion in the antibiotic resistance marker *tetA(Z)*. The first change, GabD^P134L^, accelerated growth and increased the glutarate titer by about two fold to 0.12 ± 0.00 h^−1^ and 45 ± 2 mM, respectively. The second change, pEC-XT99A-*tetA(Z)*^Δ21bp^-*gabTD*^P134L^, improved the growth rate to 0.14 ± 0.00 h^−1^ (compare isogenic strains GluA RG2 and GluA RG3 in Fig. 4). Although the glutarate titer was not increased, the volumetric productivity was increased (Fig. 4). 5AVA remained a by-product of accelerated, flux enforced glutarate production. In the fermentations performed in batch and fed-batch mode, it was demonstrated that as long as residual glucose is present 5AVA accumulated, but once it is negligible, only glutarate accumulated. *C. glutamicum* possesses the native operon *gabTDP* on its chromosome and its expression is reduced in the presence of glucose, gluconate, and *myo*-inositol, presumably via the cAMP-dependent global regulator GlxR, for which a binding site is present downstream of the *gabT* transcriptional start site [66]. Thus, while plasmid-borne expression of *ldcC*, *patD* and *patA* was sufficient for conversion of lysine to 5AVA, plasmid-borne expression of *gabTD* was limiting for conversion of 5AVA to glutarate and glutarate production benefitted from expression of the native *gabTDP* from the chromosome. Since flux enforcement by *gdh* deletion is more efficient when coupled to one transamination reaction rather than to two [8], either pathways with just one transamination reaction shall be used or flux enforcement has to be accentuate further, e.g., by combined deletion or attenuation of *gdh* and *gltBD*.

With the aim to ensure appropriate industrially applicable purification, reactive extraction followed by a reactive re-extraction step proved to be a successful recovery strategy for glutarate produced by fermentation. Reactive extraction systems containing any amount of T-C6 or high amounts of T-C8 (molar ratio of T-C8/glutarate = 6/1 or higher) led to the formation of a third solid phase. Increasing the polarity of the organic phase by addition of 1-dodecanol as (polar) modifier successfully prevented the third-phase formation. An optimal reactive extraction system was identified containing T-C6 as amine extractant at a molar ratio of 9/1 including 10 wt% of 1-dodecanol as polar modifier in the organic phase. Within these experiments, a yield of 58.1% was achieved. Only small amounts of l-glutamic acid and 5-aminovalerate were co-extracted within this step. Reactive re-extraction using both WSA’s led to maximum re-extraction yield of 99%.

One reason that a full recovery of glutarate from fermentation broth could not be achieved in the initial reactive extraction step is the presence of strong electrolytes like chloride ions in the aqueous phase, which significantly lowers the reactive extraction performance [41, 67]. Other acids as e.g. hydrochloric acid can form complexes with the amine extractant as well, hence competing with the carboxylic acid for the amine extractant and lowering reactive extraction yield [67, 68]. Previous studies showed that the effect of sulfate ions on reactive extraction yield of carboxylic acids is less pronounced [41, 67]. Therefore, choosing sulfuric acid in this study to adjust the pH of the aqueous phase before reactive extraction was beneficial for the efficiency of the process. Furthermore, it was shown in experiments investigating reactive extraction of carboxylic acids from fermentation broth, that exchanging the ammonium source NH_4_Cl for ammonium sulfate ((NH_4_)_2_SO_4_) did increase the extraction yield significantly [41]. Therefore, this approach could lead to higher extraction yields for glutarate as well and should be considered in future studies as omitting chloride in CGXII did not affect glutarate production in the evolved strain (Additional file 1: Figure S3). In conclusion, recovery of glutarate from fermentation broth applying the reactive extraction/reactive re-extraction concept for the purification of carboxylic acids was successful, hence adding to the feasibility of the industrial applicability of glutarate production by *C. glutamicum*.

## Material and methods

### Microorganisms and cultivation conditions

*E. coli* strain DH5α was used as a cloning host [69], grown in lysogeny broth (LB) at 37 °C and supplemented with appropriate antibiotics (25 μg mL^−1^ kanamycin, 100 μg mL^−1^ spectinomycin, 5 μg mL^−1^ tetracycline). *C. glutamicum* ATCC 13032 derived strains were cultivated in brain heart infusion with 0.5 M sorbitol (BHIS), supplemented with 25 μg mL^−1^ kanamycin, 100 μg mL^−1^ spectinomycin, 5 μg mL^−1^ tetracycline if appropriate. All bacterial strains and plasmids are listed in Tables 2 and 3. Growth experiments with *C. glutamicum* were performed in CGXII minimal medium [70] supplemented with 40 g L^−1^ glucose as sole carbon source and induced with 1 mM IPTG. Overnight cultures in 10 mL BHIS supplemented with the respective antibiotics were harvested and washed twice in TN buffer (50 mM Tris–HCl, 50 mM NaCl, pH 6.3) before inoculation to an initial OD_600_ of 1. The cultivations in the BioLector microfermentation system (m2p-labs, Baesweiler, Germany) were performed in 3.2 mL FlowerPlates at 1100 rpm and 30°C with filling volume of 1000 μL. To omit chloride from the culture medium, CaCl_2_ × 2H_2_O was substituted in equimolar amounts by calcium acetate, whereas NiCl_2_ × 6H_2_O was replaced by NiSO_4_. For the titration of tetracycline the washed cells were inoculated to an initial OD_600_ of 0.5 and the cultivation was performed in BHIS containing increasing concentrations of tetracycline (0, 0.3125, 0.625, 1.25, 2.5, 5, 10, 20, 40, 80 µg mL^−1^) in 10 mL Duetz microcultivation plates (Kuhner Shaker GmbH, Herzogenrath, Germany) with a culture volume of 3 mL at 220 rpm in an Ecotron ET25-TA-RC (Infors HT, Einsbach, Germany). Growth was monitored by determination of the OD_600_ with a V-1200 Spectrophotometer (VWR, Radnor, PA, USA).

**Table 2:** Bacterial strains used in this study

Strain	Relevant characteristics	References
*E. coli* DH5α	∆*lac*U169 (φ80*lac*Z ∆M15), *sup*E44, *hsd*R17, *rec*A1, *end*A1, *gyr*A96, *thi*-1, *rel*A1	[69]
*E. coli* S17-1	*recA*, *pro*, *hsdR*, RP4- 2Tc∷Mu Km∷Tn7 integrated into the chromosome	[71]
*C. glutamicum* GRLys1 (DM1933ΔCGP123)	*C. glutamicum* ATCC 13032 with modifications: Δ*pck*, *pyc*^P458S^, *hom*^V59A^, 2 copies of *lysC*^T311I^, 2 copies of *asd*, 2 copies of *dapA*, 2 copies of *dapB*, 2 copies of *ddh*, 2 copies of *lysA*, 2 copies of *lysE*, in-frame deletion of prophages CGP1 (cg1507-cg1524), CGP2 (cg1746-cg1752) and CGP3 (cg1890-cg2071)	[72]
GRLys1Δ*gltB*	GRLys1 with in-frame deletion: *gltB* (cg0229)	This study
GSLA2	GRLys1 with in-frame deletions: *sugR* (cg2115), *ldhA* (cg3219), *snaA* (cg1722), *cgmA* (cg2893)	[16]
GSLA2G	GSLA2 with in-frame deletion: *gdh* (cg2280)	[16]
GSLA2::*gltB*^E686Q^	GSLA2 carrying the mutation *gltB*^E686Q^	This study
GSLA2G::*gltB*^E686Q^	GSLA2G carrying the mutation *gltB*^E686Q^	This study
GluA	GSLA2G (pVWEx1-*ldcC*) (pEKEx3-*patDA*) (pEC-XT99A-*gabTD*)	[16]
GluA T0	GSLA2G (pVWEx1-*ldcC*) (pEKEx3-*patDA*) (pEC-XT99A-*gabTD*^P134L^)	[73]
GluA T5	Strain evolved from GluA T0 after five transfers	This study
GluA T7	Strain evolved from GluA T0 after seven transfers	This study
GluA RG1	GSLA2G (pVWEx1-*ldcC*) (pEKEx3-*patDA*) (pEC-XT99A-*tetA(Z)*^Δ21bp^-*gabTD*^P134L^)	This study
GluA RG2	GSLA2G::*gltB*^E686Q^ (pVWEx1-*ldcC*) (pEKEx3-*patDA*) (pEC-XT99A-*gabTD*^P134L^)	This study
GluA RG3	GSLA2G::*gltB*^E686Q^ (pVWEx1-*ldcC*) (pEKEx3-*patDA*) (pEC-XT99A-*tetA(Z)*^Δ21bp^-*gabTD*^P134L^)	This study

**Table 3:** Plasmids used in this study

Plasmid	Relevant characteristics	References
pEC-XT99A	Tet^R^, *C. glutamicum*/*E. coli* shuttle vector (P_trc_*, lacI*^*q*^, pGA1 o*riV*_*Cg*_)	[74]
pEC-XT99A-*gabTD*	pEC-XT99A expressing *gabT* and *gabD* from *P. stutzeri* ATCC 17588	[16]
pEC-XT99A-*gabTD*^P134L^	pEC-XT99A expressing *gabT* and *gabD* with SNP P134L from *P. stutzeri* ATCC 17588	[73]
pEC-XT99A-*tetA(Z)*^Δ21bp^-*gabTD*^P134L^	pEC-XT99A with 21 bp off-frame deletion in *tetA(Z)* expressing *gabT* and *gabD* with SNP P134L from *P. stutzeri* ATCC 17588	This study
pEKEx3	Spec^R^, *C. glutamicum*/*E. coli* shuttle vector (P_tac_ *lacI*^*q*^ pBL1, o*riV*_*Ec*_)	[75]
pEKEx3-*patDA*	pEKEx3, expressing *patD* and *patA* from *E. coli* MG1655	[16]
pEKEx3-*gltBD*	pEKEx3, expressing *gltBD* from *C. glutamicum* ATCC 13032	This study
pEKEx3-*gltB*^E686Q^*D*	pEKEx3, expressing *gltBD* with an amino acid exchange *gltB*^E686Q^ from *C. glutamicum* ATCC 13032	This study
pVWEx1	Kan^R^, *C. glutamicum*/*E. coli* shuttle vector (P_tac_, *lacI*^*q*^)	[76]
pVWEx1-*ldcC*	pVWEx1 expressing *ldcC* from *E. coli* MG1655	[77]
pK19*mobsacB*	Kan^R^, mobilizable *E. coli* vector mutagenesis (*oriV*, *sacB*)	[78]
pK19*mobsacB*-*gltB*^E686Q^	pK19*mobsacB* to introduce SNP *gltB*^E686Q^	This study
pK19*mobsacB*-Δ*gltB*	pK19*mobsacB* for deletion of *gltB* from *C. glutamicum* ATCC 13032	This study

### ALE experiments

ALE of *C. glutamicum* strain GluA T0 was carried out in repeated batch cultivations. Therefore, the replicate with the highest cell density in the exponential phase was transferred to fresh CGXII minimal medium supplemented with 40 g L^−1^ glucose as a sole carbon source and 1 mM IPTG for induction with an initial OD_600_ of 1. The experiment was performed in 10 mL Duetz microtiter plates (MTPs) with culture volumes of 3 mL at 220 rpm and 30°C in an Ecotron ET25-TA-RC (Infors HT, Einsbach, Germany). Growth was monitored by determination of the OD_600_ with a V-1200 Spectrophotometer (VWR, Radnor, PA, USA).

### Molecular biology methods

Isolation of genomic DNA of *C. glutamicum* and classical methods which include plasmid isolation, molecular cloning and heat-shock transformation of *E. coli* and electroporation of *C. glutamicum* were performed as described previously [71, 79]. ALLin HiFi DNA Polymerase (HighQu, Kraichtal, Germany) was used to amplify DNA sequences with genomic DNA as template. The oligonucleotides which were used as primers in this study are listed in Table 4. For the construction of the deletion vector the genomic flanking regions of *gltB* were amplified from genomic DNA of *C. glutamicum* ATCC13032 using the primer pairs JJ49/JJ50 and JJ51/JJ52. The PCR products were purified and linked by crossover PCR and the resulting product was cloned as XbaI/HindIII restriction site in pK19*mobsacB*. For introduction of the point mutation, plasmid pK19*mobsacB* [78] digested with BamHI was assembled with amplified DNA fragments flanking 500 bp upstream and downstream the mutation site of the gene *gltB* (cg0229) using Gibson Assembly. The SNP was introduced over the primers GltBB and GltBC. The constructed suicide vectors were transferred into *E. coli* S17-1 to follow a protocol for gene deletion routinely applied [70]. For the construction of pEKEx3-*gltBD* and pEKEx3-*gltB*^E686Q^*D* the DNA sequence was amplified from the genomic DNA of *C. glutamicum* ATCC13032 and GSLA2G::*gltB*^E686Q^, respectively. The amplified genes were assembled with pEKEx3 digested with BamHI using Gibson assembly.

**Table 4:** Oligonucleotides used as primers in this study

Primer	Sequence (5′–3′)
JJ49	GGC**AAGCTT**ATCCGTCCCAGTGGGCCT
JJ50	CCCATCCACTAAACTTAAACAGAGTCCTTGTGGTTTCAT
JJ51	TGTTTAAGTTTAGTGGATGGGGACCCAGCAATCAAGATCATGGAGGCAGTGAGCTAA
JJ52	CGG**TCTAGA**TGCACCCAGCCTTCGCGG
JJ108	CATTTGGAACCGGCATGTCCC
JJ109	GGTGCCGGTTGCGAGGAGGATC
GltBA	*GCATGCCTGCAGGTCGACTCTAGAG*CACCGTTGGACTCTATCCG
GltBB	TCGATGGTTTGAAATGCCATG
GltBC	CATGGCATTTCAAACCATCGA
GltBD	*AATTCGAGCTCGGTACCCGGGGATC*CTGAACTCAAACAGTCCACG
GltBE	CAACTACATGGCGCACTCTC
GltBF	GTTCCACATCAAATCGGCGG
gltB-for	CCTGCAGGTCGACTCTAGAGGATTCCGAAAGGAGGCCCTTCAGATGAAACCACAAGGAC
gltB-rev	TTGATGAATCCTTGTGGGTCGGCCATTAGCTCACTGCCTC
gltD-for	CAAGATCATGGAGGCAGTGAGCTAATGGCCGACCCACAAG
gltD-rev	AGTGAATTCGAGCTCGGTACCCGGGCTAGACAGCCAGCGG

Overlaps to the vector are indicated in italics, nucleotides for amino acid exchanges are underlined, restriction sites are marked in bold

### Quantification of amino acids, carbohydrates and organic acids by HPLC

The quantification of extracellular amino acids and their derivatives, carbohydrates and carboxylic acids in the cultivation medium was performed with a high-performance liquid chromatography system (1200 series, Agilent Technologies Deutschland GmbH, Böblingen, Germany). After centrifugation of 1 mL of cell cultures at 14,000 rpm for 10 min the supernatant was stored at − 20 °C prior to analysis. Analysis of l-lysine, 5AVA and the diamine cadaverine was performed by an automatic pre-column derivatization with *ortho*-phthaldialdehyde (OPA) and separated on a reversed phase HPLC using pre- and main column (LiChrospher 100 RP8 EC-5μ, 125 mm × 4.6 mm, CS Chromatographie Service GmbH) with l-asparagine as internal standard [80]. The elution buffer gradient consisted of 0.25% Na-acetate (pH 6.0), as the polar phase and methanol as the nonpolar phase [77]. Detection of the fluorescent derivatives was carried out with a fluorescence detector with an excitation wavelength of 230 nm and an emission wavelength of 450 nm. Glutarate and glucose concentrations were measured with an amino exchange column (Aminex, 300 mm × 8 mm, 10 μm particle size, 25 Å pore diameter, CS Chromatographie Service GmbH) under isocratic conditions with 5 mM sulphuirc acid as described previously with a flow of 0.8 mL min^−1^ [81]. The substances were detected with a refractive index detector (RID G1362A, 1200 series, Agilent Technologies) and a diode array detector (DAD G1315B, 1200 series, Agilent Technologies) at 210 nm.

### Coupled in vitro activity of GabT and GabD

The apparent activities of GABA transaminase GabT and succinate semialdehyde oxidoreductase GabD were assayed in combination by monitoring NADPH formation after the addition of 5AVA. Pellets from *E. coli* strains were obtained from cultivations in 50 mL LB supplemented with 1 mM IPTG and 5 µg mL^−1^ tetracycline. The pellets were washed in 20 mL 50 mM phosphate buffer (pH 7.0) and centrifuged for 10 min at 4000 rpm and 4 °C, and resuspended in 1 mL of lysis buffer (50 mM phosphate buffer pH 7.0 with 9% glycerol and 1 mM DTT). Cells were disrupted by sonication (cycle. 0.5, amplitude of 55%, on ice) for 2 min. To remove cells debris, centrifugation was performed for 1 h at 14,000 rpm and 4 °C. The supernatant was used for measuring the apparent enzyme activities. The 1 mL assay mix contained 150 mM phosphate buffer (pH 9.0), 0.1 mM pyridoxal-5′-phophate, 1 mM NADP^+^, 15 mM 2-oxoglutarate, and 0.5 mg mL^−1^ crude extract. To compare the different GabD variants, glutarate was added in different concentrations of 0 mM, 10 mM, 20 mM, 30 mM and 40 mM, respectively. The reaction was started by the addition of 20 mM 5AVA. Protein concentrations were determined with the Bradford assay kit (Bio-Rad Laboratories, Hercules, CA, United States) using BSA (bovine serum albumin) as standard. The formation of NADPH was monitored photometrically at 340 nm and 30 °C for 3 min using a Shimadzu UV-1202 spectrophotometer (Shimadzu, Duisburg, Germany).

### In vitro activity of GltBD

The apparent activity of GOGAT at different glutamine concentrations was assayed by monitoring NADP^+^ formation after the addition of glutamine. Pellets of *C. glutamicum* strains were harvested from a 50 mL CGXII minimal culture supplemented with 40 g L^−1^ glucose, 1 mM IPTG and appropriate antibiotics in the exponential phase. The pellets were washed with 20 mL 100 mM phosphate buffer (pH 7.0) and centrifuged for 10 min at 4000 rpm and 4 °C, and resuspended in 2 mL of 100 mM phosphate buffer (pH 7.0). Cells were disrupted by sonication (cycle. 0.5, amplitude of 55%, on ice) for 9 min. To remove cells debris, centrifugation was performed for 1 h at 14,000 rpm and 4 °C. The supernatant was used for measuring the apparent enzyme activities. The 1 mL assay mix contained 100 mM phosphate buffer (pH 7.0), 1.5 mM NADPH, 35 mM 2-oxoglutarate, and 0.5 mg mL^−1^ crude extract [82]. To determine the *K*_m_ glutamine was added in different concentrations of 0 mM, 0.1 mM, 0.25 mM, 0.5 mM, 1 mM, 2 mM, 3.5 mM, 5 mM and 10 mM, respectively. The reaction was started by the addition of glutamine. Protein concentrations were determined with the Bradford assay kit (Bio-Rad Laboratories, Hercules, CA, United States) using bovine serum albumin (BSA) as standard. The formation of NADP^+^ was monitored photometrically at 340 nm and 30 °C for 3 min using a Shimadzu UV-1202 spectrophotometer (Shimadzu, Duisburg, Germany).

### Fermentative production

A baffled bioreactor with total a volume of 3.7 L was used (KLF, Bioengineering AG, Switzerland). Three six-bladed Rushton turbines were placed on the stirrer axis with a distance from the bottom of the reactor of 6, 12, and 18 cm. The aspect ratio of the reactor was 2.6:1.0 and the stirrer to reactor diameter ratio was 0.39. Automatic control of the stirrer speed between 400 and 1500 rpm kept the relative dissolved oxygen saturation at 30%. A constant airflow of 2 NL min^−1^ was maintained from the bottom through a sparger, corresponding to an aeration of 1 vvm. The pH was kept constant at 7.0 ± 0.1 by automatic addition of phosphoric acid (10% (*v/v*)) and potassium hydroxide (4 M). The temperature was maintained at 30 °C. To prevent foaming 0.6 mL L^−1^ of the antifoam agent AF204 (Sigma Aldrich, Darmstadt, Germany) was added and a mechanical foam breaker was present to serve as an additional foam control. The initial working volume of 2 L was inoculated to an OD_600_ of 1.2 from a shake flask pre-culture in CGXII minimal medium supplemented with 40 g L^−1^ glucose and 1 mM IPTG. Samples were collected by an autosampler and cooled down to 4 °C until further use.

The fed-batch fermentation was performed with a head space overpressure of 0.4 bar and 42 g L^−1^ MOPS was added to the medium. The feed (ρ = 1.1 ± 0.0 kg m^−3^) consisted of 150 g L^−1^ glucose, 40 g L^−1^ (NH_4_)_2_SO_4_, 1 mL L^−1^ PCA-solution (30 mg mL^−1^ of 3,4-dihydroxybenzoic acid), 0.55 mL L^−1^ of filtered FeSO_4_-citrate solution (20 g L^−1^ FeSO_4_ heptahydrate and 20.2 g L^−1^ citrate monohydrate), 0.4 mL L^−1^ filtered vitamin solution (0.3 g L^−1^ biotin, 0.5 g L^−1^ thiamin hydrochloride, 2 g L^−1^ calcium pantothenate, and 0.6 g L^−1^ nicotinamide) and 1 mM IPTG as described before [16, 83]. It was started when the pO_2_ fell below 30% for the first time. The feed solution was applied when the pO_2_ surpassed 60%. Further feed was only added, when the pO2 decreased to 30% after addition of the feed solution to prevent oversaturation with glucose.

### Whole-genome sequencing

For isolation of genomic DNA (gDNA), *C. glutamicum* GluA T0 and the evolved strains GluA T5 and GluA T7 were cultivated in triplicates in BHIS in 100 mL baffled shake flasks at 120 rpm and 30 °C overnight. 10 mL of the cultures were harvested and gDNA was isolated using the NucleoSpin Microbial DNA kit for DNA, RNA and protein purification (Macherey–Nagel, Düren, Germany) according to the manufacturer’s manual.

Whole-genome sequencing was performed with isolated gDNA from the originating strain *C. glutamicum* GluA T0 and the evolved strains GluA T5 and GluA T7. Quality of isolated gDNA was analyzed using a spectrophotometer (NanoDrop®, ND-1000). The Illumina TruSeq DNA PCR-free high-throughput library prep kit (Illumina Inc.) was used according to manufactures instructions and Illumina genome sequencing was performed with a HiSeq1500 sequencer system 2 × 250 nt PE v2 HT rapid mode with 0.5% flow cell loading per sample (Illumina, San Diego, USA). The raw sequencing data are available via BioProject: PRJNA691520. Trimming and mapping of NGS raw reads was performed with Bowtie2 [84] paired end mode on the reference genome *C. glutamicum* ATCC13032 (CP025533) and plasmids (pVWEx1-*ldcC*; pEKEx3-*patDA*; pEC-XT99A-*gabTD*^P134L^) [13] using standard settings. Mapped sequencing data was imported in the software readXplorer v.2.2.3 [85] for visualization and SNP detection. SNP detection in all coding sequences of *C. glutamicum* was performed in readXplorer using 90% minimum percentage of variation and a minimum of 20 Reads as thresholds. Additionally, genomic DNA and plasmids of the strains were sequenced using Nanopore sequencing technology (Oxford Nanopore Technologies Oxford, UK [ONT]). Libraries were prepared with ONT SQK-LSK109 ligation sequencing kit and long read sequencing was performed on the ONT GridION platform with an R9.4.1 flow cell. Base calling and demultiplexing were performed using Guppy v3.1.5. Nanopore data were processed with Canu v1.8 [86] (parameters: genomeSize = 3.5 m, rawErrorRate = 0.3, correctedErrorRate = 0.1). Canu contigs were polished with Racon v1.3.3 [87] (parameters: − c 6, − m 8, − x − 6, − g − 8, − w 500), followed by medaka v0.11.0 [88] (parameters: − b 100, − m r941_min_high_g303) and Pilon v1.22 [89]. Unicycler v0.4.6 [90] was used for hybrid assembly of the Illumina data and the contigs from the polished Canu assembly. The unicycler assemblies were then analyzed via mauve [91] used for genome comparisons.

### RNA isolation, cDNA library preparation and sequencing

To explore differences in the gene expression in the evolved strains, *C. glutamicum* GluA T0, GluA T5 and GluA T7 were grown in CGXII minimal medium with 40 g L^−1^ glucose supplemented with 1 mM IPTG using three glycerol stocks of the respective strains. 1 mL of exponentially growing cells (OD_600_ ~ 6.0) were harvested by centrifugation (14,000×*g*, 1 min) and kept at − 80 °C. RNA isolation, purification and quality control was performed as described [92] and the high quality RNA (RNA integrity number > 9.0) was kept at − 80 °C until further use. Ribo-Zero rRNA Removal Kit (Bacteria) from Illumina (San Diego, CA, USA) was used to remove the ribosomal RNA molecules from the isolated total RNA. Preparation of cDNA libraries were performed according to the manufacturer’s instructions of TruSeq stranded mRNA Kit (Illumina, San Diego, USA). Subsequently, each cDNA library was sequenced on a HiSeq1500 (2 × 70 nt PE rapid v2) and NextSeq 500 (2 × 75 nt PE mid output v2.5) Sequencer system (Illumina, San Diego, USA). The software Bowtie2 [84] was used for mapping to the respective genome *Corynebacterium glutamicum* ATCC13032 (CP025533) and plasmids (pVWEx1-*ldcC*; pEKEx3-*patDA*; pEC-XT99A-*gabTD*^P134L^) [13]. In order to perform differential gene expression analysis DEseq2 [93] was used as a module of the software ReadXplorer(2). Statistically significant expression changes with an adjusted p-value ≤ 0.05 and a log2 fold change > 1.0 or < − 1.0 were regarded as true. The transcriptomic data have been deposited in the ArrayExpress database at EMBL-EBI1 under accession number E-MTAB-10025.

### Reactive extraction of glutarate

A detailed description of the applied method for the recovery of carboxylic acids from fermentation broth via complex formation with amine extractants can be found in literature [41, 55, 94]. The following is a brief description: using water immiscible amine extractants in a hydrophobic organic solvent, the undissociated carboxylic acid is first extracted (via complex formation) into an organic phase at a pH value of the fermentation broth which is lower than the pK_a_ of the acid. Subsequently, the carboxylic acid is re-extracted into an aqueous phase via complex formation with water-soluble amine extractants. In the case that a third, solid phase is formed because of a low polarity of the organic phase, a modifier can be added to the organic phase in order to increase the polarity and thus prevent third-phase formation.

In this work, tri-n-hexylamine (T-C6, 96% purity), purchased from Sigma-Aldrich (St. Louis, MO, USA) and tri-n-octylamine (T-C8, 97% purity), purchased from TCI (Tokyo, Japan), were applied to recover glutarate from the fermentation broth (extraction step). For re-extraction, the water-soluble primary amines *n*-propylamine (M-C3, 98% purity) from Sigma-Aldrich and n-butylamine (M-C4, 99% purity) purchased from AlfaAesar (Karlsruhe, Germany) were applied as amine extractants. The ester ethyl oleate (AlfaAesar, techn., 70%) was chosen as hydrophobic organic solvent, providing the advantage of being biocompatible [55]. If necessary, 1-dodecanol (Sigma-Aldrich, 98% purity) was added as suitable modifier to the organic phase (for extraction systems with a third phase). Prior to the reactive extraction, in order to obtain glutaric acid in its undissociated form (desired for complex formation), the pH of the fermentation broth was adjusted to 2.5 (< pK_a1_ = 4.32 of glutarate) using highly concentrated sulfuric acid (98%, from Merck, Darmstadt, Germany).

Reactive extraction and reactive re-extraction experiments were conducted in 15 mL centrifugal tubes with sealed screw caps manufactured by VWR International (Radnor, PA, USA). The phase ratio was 1/1 aqueous to organic ($V_{aq} /V_{org}$) with a total volume of 5 mL (4 mL in case of re-extraction) of each experiment. The chemicals were added in the order fermentation broth, solvent, modifier (if necessary) and amine extractant (re-extraction: water, amine extractant, organic phase after reactive extraction) and each compound was weighed using a BP 301S (Sartorius, Göttingen, Germany) mass balance with an accuracy of ± 1 mg. The centrifugal tubes were then transferred to an overhead shaker (Trayster Digital from IKA, Staufen, Germany) and mixed for 24 h at *T* = 25 °C to ensure equilibration of the system. Centrifugation of the 15 mL tubes was done in a Centrifuge 5804 R Eppendorf (Hamburg, Germany) equipped with an A4-44 rotor at 3500 rpm and a temperature of *T* = 25 °C for 15 min. For analysis of the aqueous phase, a sample of the aqueous phase after reactive extraction was taken by punching a cannula through the bottom of the centrifugal tube.

Concentration analysis of the aqueous phase was performed via HPLC with an Agilent 1200 Series HPLC (Santa Clara, CA, USA) and a Nucleogel Sugar 810 H, 7.8 × 300 mm column including the corresponding guard column (both by Macherey–Nagel, Düren, Germany). The temperature was kept constant at* T* = 35 °C. 5 mM sulfuric acid was used as eluent at a volume flow of 0.6 mL min^−1^. Detection was realized with a refractive index detector. All aqueous samples were filtrated using a poly-ethersulphonate syringe filter (0.45 µm) by VWR International (Radnor, USA) and diluted with eluent at a ratio of 1/5 ($V_{aq} /V_{eluent}$) before analysis. The injected sample amount was 5 µL.

The reactive extraction yield $X_{RE}$ was calculated using Eq. 1, where $n_{GA,0}^{aq1}$ is the molar amount of glutarate in the fermentation broth before reactive extraction, whereas $n_{GA}^{aq1}$ is the molar amount of glutarate in the aqueous phase after reactive extraction.

1 $$X_{RE} = \frac{{n_{GA,0}^{aq1} - n_{GA}^{aq1} }}{{n_{GA,0}^{aq1} }} = \frac{{n_{GA}^{org} }}{{n_{GA,0}^{aq1} }}.$$

The molar amount of glutarate in the organic phase after reactive extraction $n_{GA,0}^{org}$ was calculated via mass balance of glutarate in the aqueous phase.

The re-extraction yield $X_{REEX}$ was calculated using Eq. 2, in which $n_{GA}^{aq2}$ is the molar amount of glutarate in the aqueous phase after re-extraction and $n_{GA}^{org}$ is the molar amount of glutarate in the organic phase after reactive extraction and before re-extraction.

2 $$X_{REEX} = \frac{{n_{GA,0}^{aq2} }}{{n_{GA}^{org} }}.$$

## Supplementary Information

**Additional file 1: Figure S1.** (**A**) DNA region of the plasmid pEC-XT99A-*gabTD*^P134L^ with the 21 bp sequence deleted in ALE strain GluA T7 (boxed in red). (**B**) Determination of the minimum inhibitory concentration for tetracycline for strain GluA T0 with the orginal plasmid pEC-XT99A-*gabTD*^P134L^ and GluA RG1 carrying this plasmid with the 21 bp deletion. Cells were grown in Duetz cultivation system using BHIS supplemented with increasing concentrations of tetracycline (0–80 µg µL^−1^). The ΔOD_600_ values were determined after 48 hours and are shown as means with standard deviations (n = 3 cultivations). **Figure S2.** Dependence of the specific acitvity of GOGAT on the glutamine concentration determined for GRLys1Δ*gltB* overexpressing the native version of *gltBD* (**A**) or the mutated *gltB*^E686Q^*D* (**B**). Crude extracts of GRLys1Δ*gltB* (pEKEx3-*gltBD*) and GRLys1Δ*gltB* (pEKEx3-*gltB*^E686Q^*D*) were assayed for specific activity of the glutamate synthase (GOGAT) in the presence of increasing glutamine concentrations (0 mM, 0.1 mM, 0.25 mM, 0.5 mM, 0.75 mM, 1 mM, 2 mM, 5 mM, 10 mM). Values represent means and standard deviations of triplicate measurements. **Figure S3.** Glutarate production by GluA T7 in chloride-free medium. Cells were grown in Duetz cultivation system using either standard CGXII minimal medium or CGXII minimal medium without chloride supplemented with 40 g L^−1^ glucose, induced with 1 mM IPTG, and harvested after 48 h. Values and error bars represent means and standard deviations of glutarate (green) and 5AVA concentrations (red) in the culture supernatants (n = 3 cultivations).

**Additional file 2: Table S1.** DESeq2 differential gene expression analysis of *Corynebacterium glutamicum* ALE strain GluA T5 compared with control strain GluA T0. **Table S2.** DESeq2 differential gene expression analysis of *Corynebacterium glutamicum* ALE strain GluA T7 compared with control strain GluA T0.

## Data Availability

All data generated or analysed during this study are included in this published article and its additional files. The mapped genome sequencing data is available via BioProject: PRJNA691520 and the transcriptomic data is available via the ArrayExpress database at EMBL-EBI1 under Accession Number E-MTAB-10025.
