# Supplementary material for: Adaptive laboratory evolution accelerated glutarate production by Corynebacterium glutamicum
Source: Microb Cell Fact. 2021 May 10;20:97. doi: 10.1186/s12934-021-01586-3 (PMC8112011; doi:10.1186/s12934-021-01586-3)
Supplement: Supplementary file 1 — Additional file 1: Figure S1. (A) DNA region of the plasmid pEC-XT99A-gabTDP134L with the 21 bp sequence deleted in ALE strain GluA T7 (boxed in red). (B) Determination of the minimum inhibitory concentration for tetracycline for strain GluA T0 with the orginal plasmid pEC-XT99A-gabTDP134L and GluA RG1 carrying this plasmid with the 21 bp deletion. Cells were grown in Duetz cultivation system using BHIS supplemented with increasing concentrations of tetracycline (0–80 µg µL−1). The ΔOD600 values were determined after 48 hours and are shown as means with standard deviations (n = 3 cultivations). Figure S2. Dependence of the specific acitvity of GOGAT on the glutamine concentration determined for GRLys1ΔgltB overexpressing the native version of gltBD (A) or the mutated gltBE686QD (B). Crude extracts of GRLys1ΔgltB (pEKEx3-gltBD) and GRLys1ΔgltB (pEKEx3-gltBE686QD) were assayed for specific activity of the glutamate synthase (GOGAT) in the presence of increasing glutamine concentrations (0 mM, 0.1 mM, 0.25 mM, 0.5 mM, 0.75 mM, 1 mM, 2 mM, 5 mM, 10 mM). Values represent means and standard deviations of triplicate measurements. Figure S3. Glutarate production by GluA T7 in chloride-free medium. Cells were grown in Duetz cultivation system using either standard CGXII minimal medium or CGXII minimal medium without chloride supplemented with 40 g L−1 glucose, induced with 1 mM IPTG, and harvested after 48 h. Values and error bars represent means and standard deviations of glutarate (green) and 5AVA concentrations (red) in the culture supernatants (n = 3 cultivations). [file 12934_2021_1586_MOESM1_ESM.docx]

**Additional file 1 to**

Adaptive Laboratory Evolution accelerated glutarate production by *Corynebacterium glutamicum*

Carina Prell^1^, Tobias Busche^2^, Christian Rückert^2^, Lea Nolte^3^, Christoph Brandenbusch^3^ and Volker F. Wendisch^1,^*

^1^ Genetics of Prokaryotes, Faculty of Biology & CeBiTec, Bielefeld University, Universitätsstr. 25, 33615 Bielefeld, Germany; [carina.prell@uni-bielefeld.de](mailto:carina.prell@uni-bielefeld.de)

^2^ Technology Platform Genomics, Center for Biotechnology (CeBiTec), Bielefeld University, Sequenz 1, 33615 Bielefeld, Germany; [tbusche@cebitec.uni-bielefeld.de](mailto:tbusche@cebitec.uni-bielefeld.de); cruecker@cebitec.uni-bielefeld.de

^3^ Laboratory of Thermodynamics, Department of Biochemical and Chemical Engineering, TU Dortmund University, Emil-Figge-Str. 70, 44227 Dortmund, Germany; [lea.nolte@tu-dortmund.de](mailto:lea.nolte@tu-dortmund.de); [christoph.brandenbusch@tu-dortmund.de](mailto:lea.nolte@tu-dortmund.de)

***** Correspondence: [volker.wendisch@uni-bielefeld.de](mailto:volker.wendisch@uni-bielefeld.de)


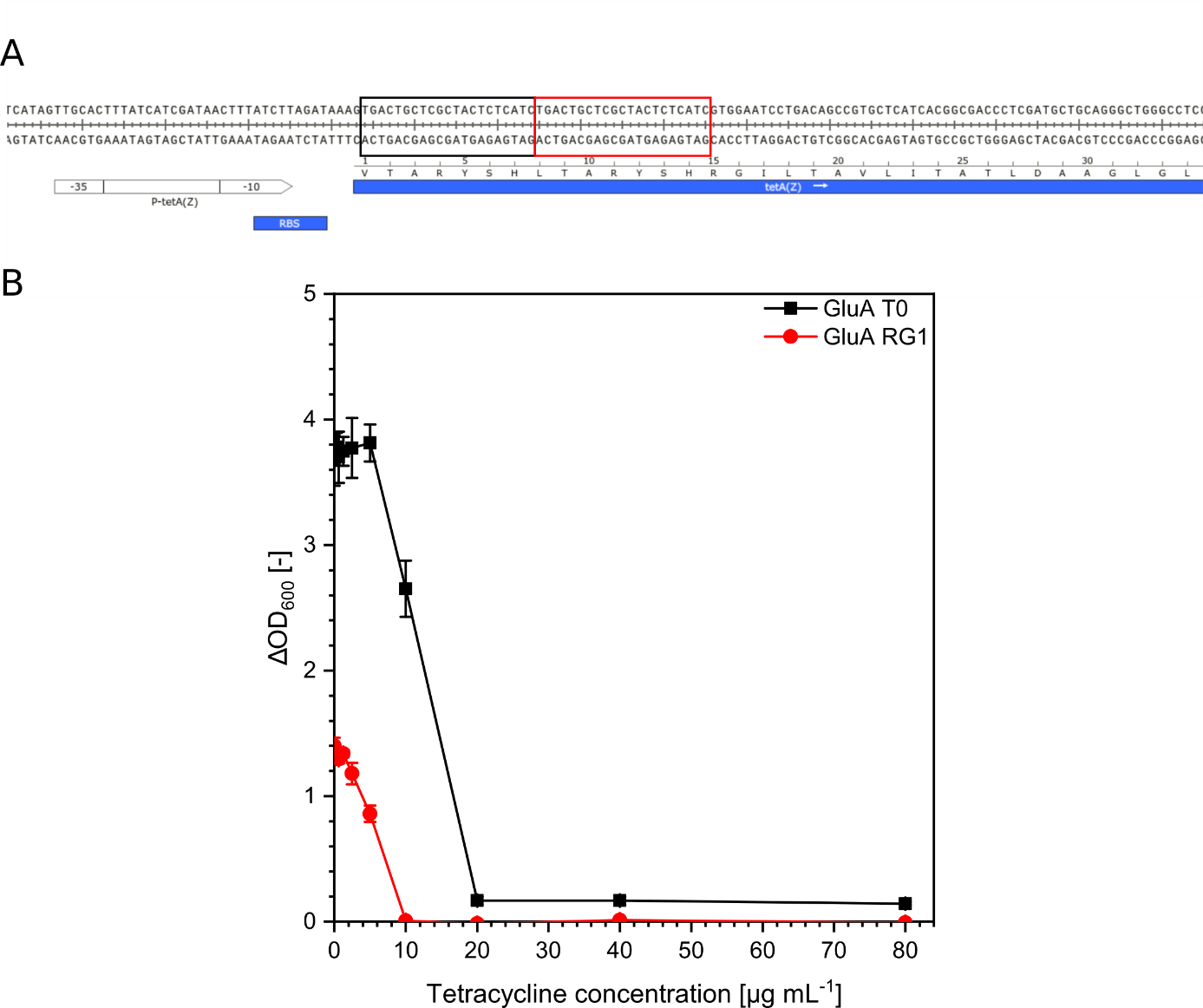


**Figure S1: (A) DNA region of the plasmid pEC-XT99A-*gabTD*^P134L^ with the 21 bp sequence deleted in ALE strain GluA T7 (boxed in red)** The DNA-sequence of the parental plasmid is partially shown including the promoter region of *tetA(Z)* and the first 102 bp of its coding sequence. The 21 bp off-frame deletion is indicated by a red box, whereas the direct repeat is marked by a black box. **B) Determination of the minimum inhibitory concentration for tetracycline for strain GluA T0 with the orginal plasmid pEC-XT99A-*gabTD*^P134L^ and GluA RG1 carrying this plasmid with the 21 bp deletion.** Cells were grown in Duetz cultivation system using BHIS supplemented with increasing concentrations of tetracycline (0-80 µg µL^-1^). The ΔOD_600_ values were determined after 48 hours and are shown as means with standard deviations (n = 3 cultivations).


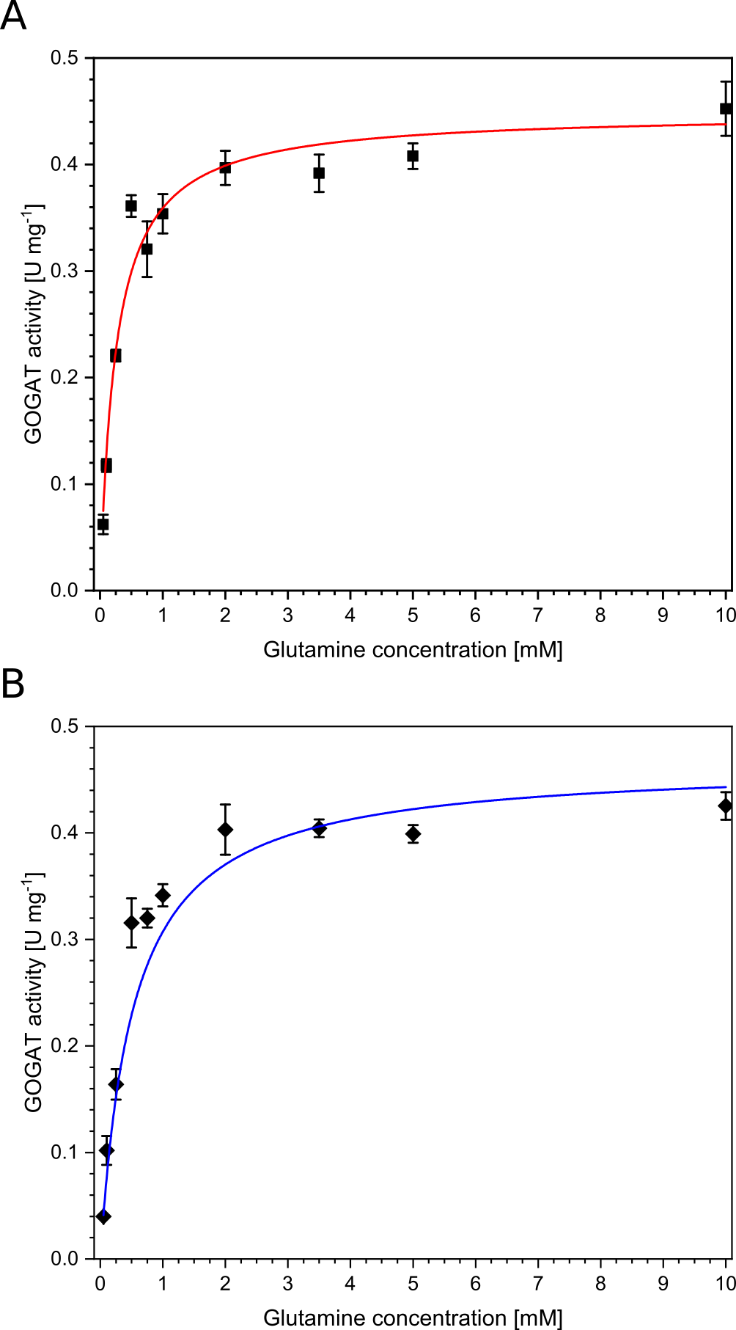


**Figure S2: Dependence of the specific acitvity of GOGAT on the glutamine concentration determined for GRLys1Δ*gltB* overexpressing the native version of *gltBD* (A) or the mutated *gltB*^E686Q^*D* (B)**. Crude extracts of GRLys1Δ*gltB* (pEKEx3-*gltBD*) and GRLys1Δ*gltB* (pEKEx3-*gltB*^E686Q^*D*) were assayed for specific activity of the glutamate synthase (GOGAT) in the presence of increasing glutamine concentrations (0 mM, 0.1 mM, 0.25 mM, 0.5 mM, 0.75 mM, 1 mM, 2 mM, 5 mM, 10 mM). Values represent means and standard deviations of triplicate measurements.


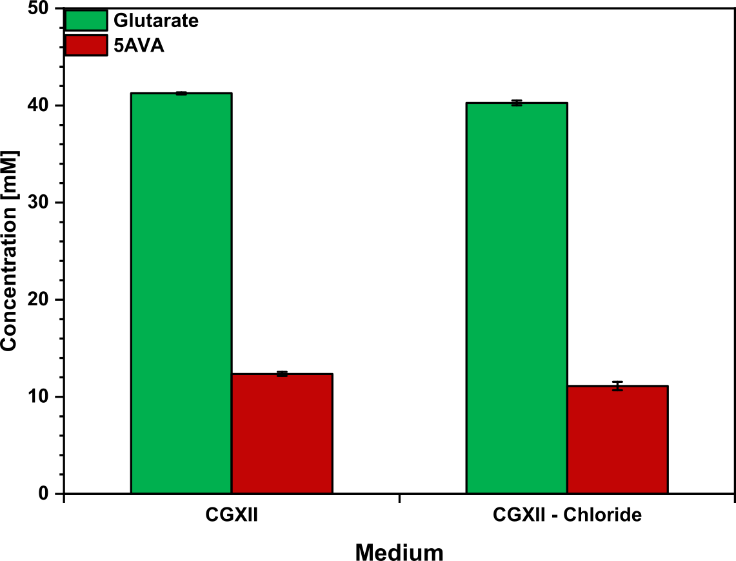


**Figure S3: Glutarate production by GluA T7 in chloride-free medium.** Cells were grown in Duetz cultivation system using either standard CGXII minimal medium or CGXII minimal medium without chloride supplemented with 4% (*w/v*) glucose, induced with 1 mM IPTG, and harvested after 48 hours. Values and error bars represent means and standard deviations of glutarate (green) and 5AVA concentrations (red) in the culture supernatants (n = 3 cultivations).
